# Advances in the biosynthesis of naturally occurring benzylisoquinoline alkaloids

**DOI:** 10.3389/fpls.2025.1548471

**Published:** 2025-01-30

**Authors:** Wanli Zhao, Jihua Liu, Yu Chen

**Affiliations:** ^1^ Jiangsu Key Laboratory for the Research and Utilization of Plant Resources, Jiangsu Province Engineering Research Center of Eco-cultivation and High-value Utilization of Chinese Medicinal Materials, Institute of Botany, Jiangsu Province and Chinese Academy of Sciences (Nanjing Botanical Garden Mem. Sun Yat-Sen), Nanjing, China; ^2^ Jiangsu Key Laboratory of Traditional Chinese Medicine. (TCM) Evaluation and Translational Research, School of Traditional Chinese Pharmacy, China Pharmaceutical University, Nanjing, China

**Keywords:** benzylisoquinoline alkaloids, biosynthesis, secondary metabolite, biosynthetic pathway, berberine bridge enzyme, cytochrome P450, methyltransferase

## Abstract

Benzylisoquinoline alkaloids (BIAs) are a prominent class of plant metabolites with significant pharmaceutical and industrial significance that have garnered substantial attention from researchers worldwide. BIAs exhibit several pharmacological activities and have been used extensively. Examples include analgesics such as morphine, tetrahydropalmatine, antimicrobials such as berberine, and antineoplastic agents including cepharanthine. Most BIAs are derived and isolated from medicinal plants; however, these plants are predominantly wild resources that are scarce. Their high environmental impact, slow growth rate, scarcity of resources, and expensive direct extraction costs pose a significant challenge. Certain BIAs are present in trace amounts in medicinal plants; moreover, they have complex chemical structures and unstable properties. Designing chemical synthesis routes and processes is challenging. Thus, a major obstacle in developing and utilizing these natural products in the pharmaceutical industry lies in their low abundance in nature. Consequently, the limited supply of these molecules fails to meet high research and market demands. In recent years, biosynthesis approaches have emerged as a novel and efficient method to obtain BIAs. In this review, recent progress in the field of enzymes related to the elucidation of biosynthetic pathways and the biosynthesis of BIAs are discussed, and future perspectives for designing viable strategies for their targeted manipulation are presented.

## 1 Introduction

**Figure 1:**
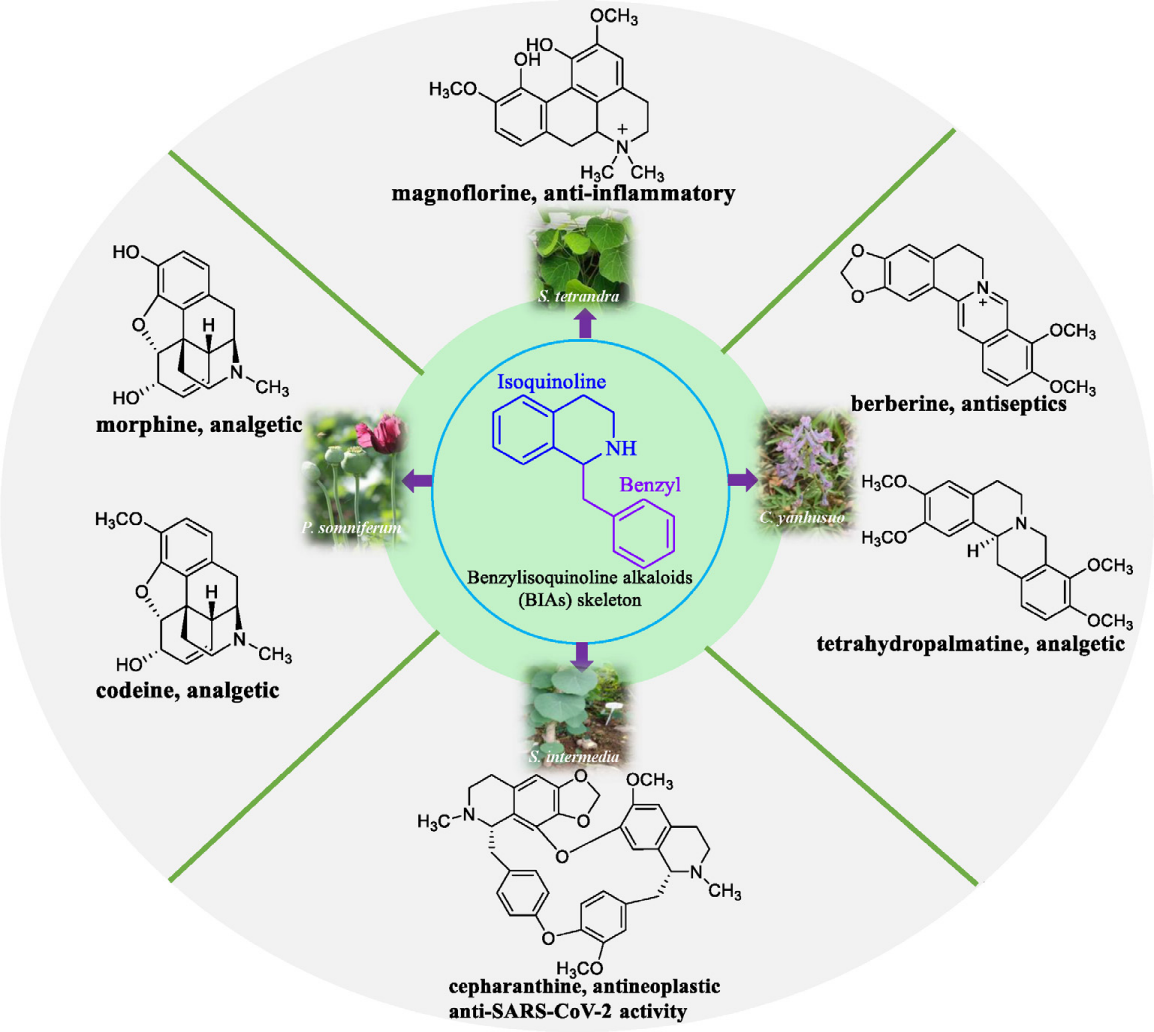
Representative compounds of BIAs and their source plants. The structure skeleton of BIA consists of an isoquinoline ring and a benzyl group. Representative BIA compounds include the analgesics morphine and codeine derived from *Papaver somniferum*, the antitumor agent cepharanthine from *Stephania intermedia*, the analgesic tetrahydropalmatine and the antibacterial agent berberine from *Corydalis yanhusuo*, and the anti-inflammatory compound magnoflorine from *Stephania tetrandra*.

Benzylisoquinoline alkaloids (BIAs) constitute a major group of alkaloids, wherein approximately 2,500 compounds have been identified (Facchini and De Luca, 2008; Minami et al., 2008; Tian et al., 2024). BIAs exhibit numerous pharmacological properties and have been used extensively. Notable examples include morphine, codeine, and tetrahydropalmatine as analgesics; berberine as an antiseptic; and cepharanthine as an antineoplastic and antiviral agent (**Figure 1**) (Hagel and Facchini, 2013; Fan et al., 2022). Currently, BIAs are predominantly found in plant families such as Galleriferaceae, Tetranyaceae, and Ranunculaceae. Most of these plants are wild resources and their growth is highly influenced by environmental factors. Moreover, they grow slowly, have limited availability, and are associated with high extraction costs. Certain BIAs are present as trace compounds in medicinal plants. They have complex chemical structures and are unstable molecules; moreover, developing synthetic processes for these compounds is challenging. In recent years, researchers have explored novel approaches to obtain naturally occurring bioactive compounds that show therapeutic potential. According to a report in the United States, approximately 25% of pharmaceuticals originate from plant-derived natural chemicals (Li et al., 2018a). However, a major obstacle in harnessing and exploiting natural products to their full potential for pharmaceutical use is their scarcity in nature, leading to an insufficient supply that fails to meet the high research and market demands. Furthermore, the complete elucidation of biosynthetic pathways for secondary plant metabolites is poorly understood. Natural products are currently isolated from plant cells or using tissue culture; however, the high costs associated with plant cell culture, the challenges in developing plant cell lines, and limited yields are some drawbacks that limit the widespread commercial application of this technique (Atanasov et al., 2015). However, advances in new generation sequencing technology, genomics, metabolomics, and bioinformatics have led to the emergence of synthetic biology as a promising approach in addressing sourcing issues related to several natural products that are of high value (Voigt, 2020; Jiang et al., 2024). The heterologous biosynthesis of naturally occurring phytochemicals in bacteria, yeast, and other host plants shows immense potential. The use of synthetic biology techniques to obtain terpenoids, alkaloids, flavonoids, and polyphenols has met with considerable success. Its efficient and environmentally friendly production chain is widely recognized by both the scientific community and the pharmaceutical industry (Paddon et al., 2013; Yao et al., 2013; Nett et al., 2023; Jiang et al., 2024). A well-defined biosynthetic pathway and the elucidation of key enzymes in these pathways serve as the foundation for BIA biosynthesis. The objective of this review was to conduct a comprehensive and systematic bibliometric analysis of the biosynthetic pathways of BIAs, elucidating key enzymes and bridging the knowledge gap in existing bibliometric reviews. We hope that this review will provide novel insights into the biosynthesis of BIAs.

## 2 Integration of multiomics approaches to elucidate the biosynthetic pathway of BIAs

**Figure 2:**
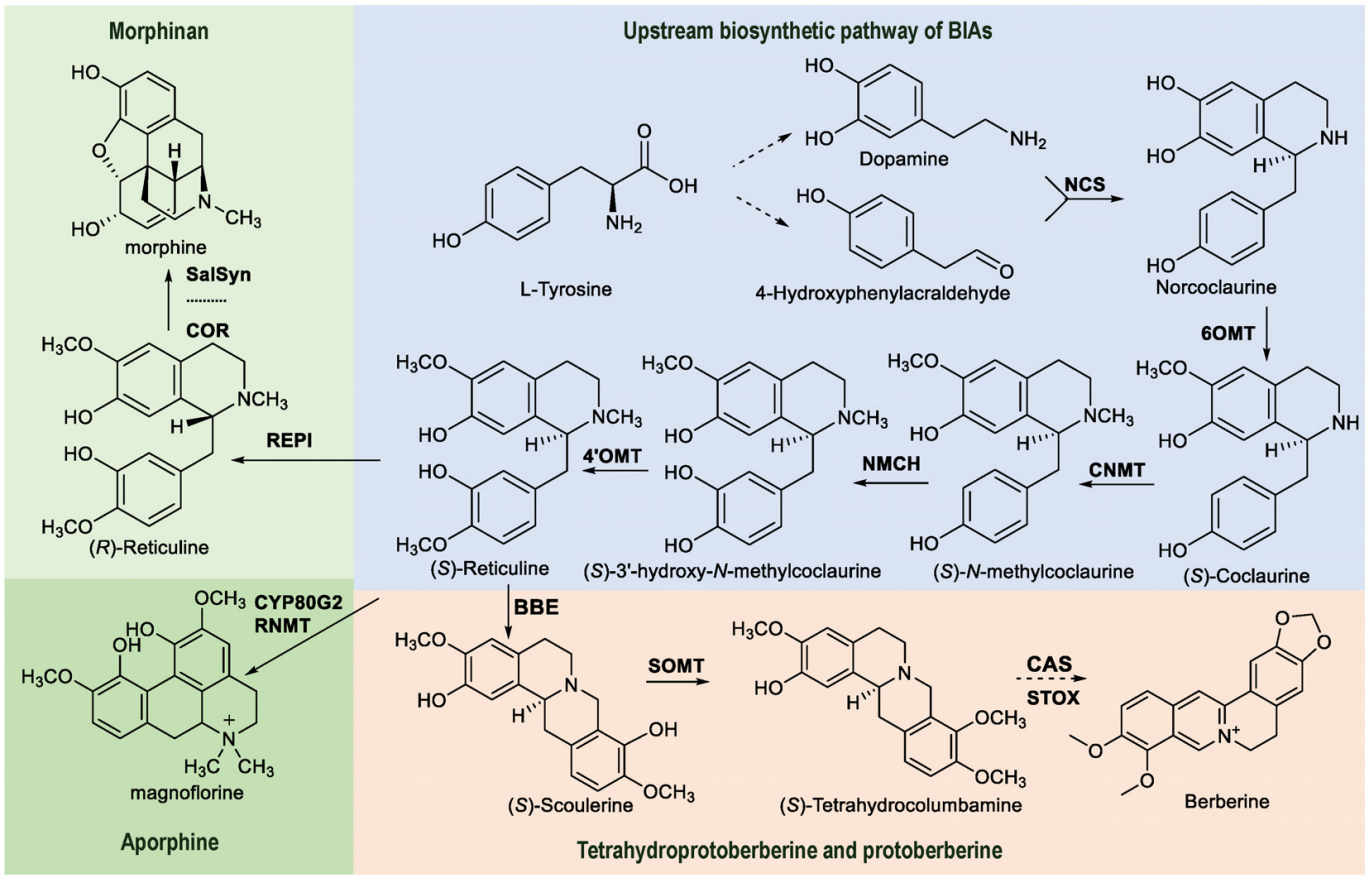
Biosynthetic pathways for the major types of BIAs, including the associated enzymes. The powder blue box represents the shared upstream biosynthetic pathway and key branch points for the synthesis of BIAs. The flesh-colored box represents the tetrahydroprotoberberine and protoberberine types; the green apple-colored box represents the morphinan type; the Maldivian green box represents the aporphine type. Norcoclaurine synthase (NCS); norcoclaurine 6-*O*-methyltransferase (6OMT); Coclaurine *N*-methyltransferase (CNMT); *N*-methylcoclaurine 3’-hydroxylase (NMCH); 4’-*O*-methyltransferase (4’OMT); Berberine bridge enzyme (BBE); (*S*)-scoulerine 9-*O*-methyltransferase (SOMT); Canadine synthase (CAS); (*S*)-tetrahydroprotoberberine oxidase (STOX); Salutaridine synthase (SalSyn); Reticuline *N*-methyltransferase (RNMT); Reticuline epimerase (REPI).

A detailed analysis of biosynthetic pathways and the study of key enzymes involved in BIA biosynthesis are essential prerequisites when using synthetic biology techniques. Furthermore, these efficient enzymes should be assembled into a biosynthetic module to construct BIA-producing engineering strains. Additionally, the screening of key enzymes and their regulatory genes in biosynthetic pathways is crucial in understanding secondary metabolic pathways. Although high-throughput sequencing enables the rapid acquisition of genome information, selecting candidate genes involved in the biosynthesis of specific natural products from numerous genes poses a challenge. Transcriptomics, metabonomics, and genomics are the primary methods currently used to screen genes that encode key enzymes in biosynthetic pathway (Zhan et al., 2022; He et al., 2023). Most BIAs share a well-defined upstream biosynthetic pathway, thereby necessitating the analysis of their downstream metabolic pathways when elucidating the biosynthetic pathway of a specific component or class. The biosynthetic pathway of BIAs utilizes L-tyrosine as its precursor, which undergoes catalysis via multiple enzymatic reaction steps to yield a series of compounds (Tan et al., 2020; Wang et al., 2022). For example, for berberine, the biosynthetic precursor tyrosine undergoes enzymatic catalysis to yield tyrosine derivatives, including dopamine and 4- hydroxyphenylacetaldehyde (**Figure 2**) (Samanani et al., 2004; Minami et al., 2007; Vimolmangkang et al., 2016). Then, dopamine and 4-hydroxyphenylacetaldehyde reacted to form norcoclaurine catalyzed by norcoclaurine synthase (NCS). The subsequent conversion of these derivatives involves norcoclaurine 6-*O*-methyltransferase (6OMT), 4′-*O*-methyltransferase (4′OMT), coclaurine *N*-methyltransferase (CNMT), and cytochrome P450 oxidoreductase (NMCH), leading to the production of (*S*)-reticuline (Ikezawa et al., 2003; Liscombe and Facchini, 2007; Khodorova et al., 2013). The next reaction is catalyzed by berberine bridge enzyme (BBE), wherein (*S*)-reticuline forms tetrahydroproberberine (*S*)-scoulerine. (*S*)-scoulerine is then catalyzed by (*S*)-scoulerine 9-*O*-methyltransferase (9OMT) to form the intermediate (*S*)-tetrahydrocolumbamine (Nakagawa et al., 2012). (*S*)-tetrahydrocolumbamine is acted upon by canadine synthase (CAS) and (*S*)-tetrahydroprotoberberine oxidase (STOX) to yield berberine (Diaz Chavez et al., 2011; Gesell et al., 2011; Hagel et al., 2012; Dang and Facchini, 2014). Reticuline serves as a pivotal intermediate and a crucial node in the biosynthetic pathways of morphinan type and aporphine type. While the upstream biosynthetic pathway of BIAs and the key enzymes that are involved are relatively well understood, there is a lack of clarity related to the specific downstream biosynthetic pathway for a particular compound. Furthermore, it is worth noting that the catalytic efficiency of a particular enzyme can vary across different plant sources. Therefore, it is crucial to analyze and elucidate the downstream biosynthetic pathways of BIAs, identify the pivotal enzymes involved in the pathway, and utilize these highly efficient synthetases for the optimal biosynthesis of these compounds.

### 2.1 Integration of transcriptomics, metabolomics and genomics to elucidate the biosynthetic pathway of BIAs

The identification and characterization of candidate enzyme genes involved in the biosynthesis of secondary metabolic products is crucial in comprehending the molecular mechanisms underlying these biosynthetic pathways (He et al., 2018). BIAs are present in various families of plants, including Papaveraceae, Ranunculaceae, Berberidaceae, and Caprophyllaceae. The biosynthetic pathways of BIAs have been extensively studied in medicinal plants such as *Papaver somniferum*, *Eschscholzia californica*, and *Coptis japonica* (Hara et al., 1994; Liang et al., 2008; Liscombe et al., 2009; Inui et al., 2012; Tekleyohans et al., 2013; Son et al., 2014; Boke et al., 2015; Agarwal et al., 2016; Purwanto et al., 2017; Oh et al., 2018). (*S*)-reticuline is a pivotal intermediate in the biosynthesis of various BIAs, facilitating the biosynthesis of diverse BIAs via the action of numerous enzymes (Farrow et al., 2012). Another crucial intermediate, (*S*)-scoulerine, is produced from (*S*)-reticuline via BBE catalysis (**Figure 2**). This key enzyme has been identified in numerous plant species (Facchini et al., 1996; Samanani et al., 2005). Furthermore, the gene for the enzyme SOMT, a crucial component in the biosynthetic pathway of BIAs, has been successfully cloned and expressed from *P. somniferum*, *Glaucium flavum*, and *C. japonica* consecutively (Morishige et al., 2010; Dang and Facchini, 2012; Chang et al., 2015). Subsequently, (*S*)-scoulerine undergoes methylation or cyclization to generate a series of BIAs. The research teams of Jillian and Facchini conducted metabolomic analysis of more than 20 BIA-producing plants using a multiplatform approach and integrating analytical methods to identify 20 nonmodel plant metabolites (Hagel et al., 2015). They also established a comprehensive compound library of related metabolites. These valuable metabolomics resources, in conjunction with genomic resources, provide favorable conditions to investigate the biosynthesis of BIAs in nonmodel plant species. *CYP80* genes involved in the biosynthesis of BIAs and aporphine were identified based on the transcriptome sequencing of lotus (*Nelumbo nucifera*) (Deng et al., 2018). The main active components of the traditional Chinese medicine Fenfangji (*Stephania tetrandra*) are BIAs. A total of 42 candidate genes involved in the 15 steps of BIA biosynthesis was discovered through metabolite and transcriptome analyses of the stems, leaves, xylem, and epidermis of *Stephania tetrandra*. Furthermore, a novel (*S*)-norcoclurine-6OMT gene was characterized for its role as a catalyst in the formation of (*S*)-coclaurine through the methylation of (*S*)-norcoclaurine *in vitro* (Li et al., 2022). Fourteen cytochrome P450s (CYP450s) and 33 methyltransferases associated with BIA biosynthesis were identified using the weighted gene co-expression network analysis of the transcriptional and metabolic profiles from 18 different tissues of *Phellodendron amurens*. Notably, a PR10/Bet v1 protein was characterized as norcoclaurine synthase, which was responsible for catalyzing the conversion of dopamine and 4-hydroxyphenylacetaldehyde into (*S*)-norcoclaurine (Liu et al., 2024a). Yang et al. identified candidate genes involved in the biosynthetic pathways of BIAs, such as sinomenine, magnoflorine, and tetrahydropalmatine from *Sinomenium acutum* through comprehensive full-length transcriptome sequencing and metabolite analysis (Yang et al., 2022). Zhao et al. elucidated the biosynthetic pathway of BIAs and identified candidate genes, including *6OMT*, *CNMT*, *NMCH*, *BBE*, *SOMT1*, *CFS*, *SPS*, *STOX*, *MSH*, *TNMT*, and *P6H* in *C. yanhusuo* through widely targeted metabolomic and transcriptomic analyses (Zhao et al., 2024). Furthermore, methyltransferases and cytochrome P450 family genes involved in BIA biosynthetic pathway were identified based on the comparative transcriptomics of *Berberis koreana*, *B. thunbergii* and *B. amurensis* (Roy et al., 2022). A combination of full-length transcriptomics and targeted metabolomics was used to identify the BIA biosynthetic genes in *Corydalis corydalis*. Ten candidate genes of columbamine-*O*-methyltransferase were screened to lay a foundation for the identification of the last key enzyme of tetrahydropalmatine in *C. corydalis* (Xu et al., 2021). The combination of full-length transcriptome sequencing and metabolomics to elucidate the biosynthetic pathways of plant secondary metabolites has attracted increasing attention due to its high efficiency and relatively low cost.

The integration of genomics, metabolomics, and transcriptomics enables determining the correlation between changes in secondary metabolites and the expression of related enzyme genes, facilitating the identification of biosynthetic genes involved in the biosynthesis of secondary metabolites and the analysis of secondary metabolic pathways. This advancement also contributes to an enhancement in the development and utilization of medicinal plant resources. Ye Kai et al. investigated the complete genome sequence of opium poppy, elucidating significant rearrangement events and duplication events that occurred during its history of evolution. Furthermore, they provided insights into the evolutionary history of gene clusters involved in the biosynthesis of morphine alkaloids in poppy, laying a crucial foundation for the further exploration of its medicinal value and unveiling the evolutionary history of Papaveraceae (Guo et al., 2018). The biosynthetic pathway genes of sanguinarine (a BIA) were successfully identified and validated by Zeng Jianguo et al. Using whole-genome sequencing of medicinal plants and construction of the *Macleaya cordata* genome map, significantly advancing the industrial production of sanguinarine (Liu et al., 2017). Cepharanthine, a class of BIAs primarily found in plants in the *Stephania* genus, has been approved by the Japanese Medicines and Medical Devices Agency to treat cancer and inflammation. It exerts anti-coronavirus effect against severe acute respiratory syndrome coronavirus-2. The genomes of *S. yunnanensis*, *S. cepharantha*, *S. japonica*, *Corydalis tomentella*, *Tinospora sagittata*, *Corydalis yanhusuo*, *Aristolochia contorta*, and *Menispermum dauricum* have been reported (Cui et al., 2022; Xu et al., 2022b; Alami et al., 2024; An et al., 2024; Leng et al., 2024; Liu et al., 2024b; Xu et al., 2024a). These genome reports provide valuable genetic resources to analyze the biosynthetic pathways of BIAs. The use of genomics has expedited the discovery of novel enzyme genes, whereas, the integration of genomics, metabolomics, and transcriptomics has provided initial insights into the regulation of BIAs. Structural analysis of key enzymes involved in BIA biosynthesis offers valuable resources for the development of biomimetic biosynthesis. However, challenges still exist in elucidating the biosynthetic pathway of BIAs, warranting further investigation to elucidate the regulatory mechanisms in plants. Due to the complexity and high costs associated with plant genome sequencing, most BIA-containing medicinal plants remain currently unexplored. With advances in sequencing technology and cost reduction, substantial genomic information on medicinal plants can be unveiled via gene sequencing.

The types and contents of BIAs from different medicinal plants exhibit significant variation. Researchers have investigated the formation mechanisms underlying the structural diversity of BIAs. A whole-genome duplication event occurred in the *Papaver somniferum* genome approximately 7.8 million years ago, while a segmental genome duplication event took place at least 110 million years ago (Guo et al., 2018). Additionally, 15 genes involved in the biosynthesis of antitussives noscapine and analgesic morphine from *P. somniferum* were identified to form supergene clusters on chromosome 11. Thus, gene duplication, rearrangement, and fusion events lead to the formation of supergene clusters that can cooperatively and efficiently synthesize specialized metabolic products, such as morphine and noscapine, in *P. somniferum*. Xu and his colleagues investigated the species evolution, whole genome duplication events, and chromosomal evolution in *Menispermum dauricum*. By comparing genomes, they identified a significant expansion of the CYP80 gene family in *M. dauricum*. This expansion, along with tissue-specific expression patterns, contributes to the diversity of BIAs. Notably, they discovered a novel enzyme MdCYP80G10 to catalyze the C2′-C4a phenol coupling of (*S*)-reticuline into sinoacutine, which is the enantiomer of morphinan compounds, with stereospecificity (An et al., 2024). Additionally, the team observed that genes encoding berberine biosynthesis in *Coptis chinensis* and other plants are dispersed across different genomic locations, whereas in *Phellodendron amurense*, enzymes involved in berberine biosynthesis, such as CYP71BG and OMT, form gene clusters (Xu et al., 2024b). These clusters underwent two species-specific whole genome duplication events, leading to the replication and neofunctionalization of key genes. Their work elucidates the convergent evolutionary mechanism of berberine biosynthesis mediated by non-homologous enzymes, offering a new paradigm for understanding how genome evolution drives plant metabolic diversity.

### 2.2 Classification and characterization of enzymes that are involved in the biosynthesis pathways of BIAs

Enzymes involved in the biosynthesis of BIAs include lyases, transferases, and redox enzymes. Lyase primarily refers to NCS, which catalyzes the condensation of dopamine and 4-hydroxyphenylacetaldehyde to generate a precursor of alkaloid biosynthesis. Methyltransferases, including *O*-methyltransferase and *N*-methyltransferase, play a crucial role in the biosynthetic pathway of BIAs. Additionally, BBE and cytochrome P450 are the main oxidoreductase enzymes that are involved.

#### 2.2.1 Methyltransferases

The methyltransferases involved in BIA biosynthesis consist of *O*- and *N*- methyltransferases. Methyltransferases play a pivotal role in secondary metabolism in plants, significantly diversifying the range of secondary metabolites through substrate methylation. Furthermore, methyltransferases guide intermediates into specific biosynthetic pathways, thereby exerting key regulatory control over the generation of secondary metabolites in plants (Morishige et al., 2010). Three crucial *O*-methyltransferases, namely 4′-*O*-methyltransferase (4′OMT), (*S*)-normonine 6OMT, and (*S*)-scoulerine 9OMT, were successively isolated and characterized in the biosynthetic pathway of BIAs (**Figure 2**) (Rueffer et al., 1983; Takeshita et al., 1995; Morishige et al., 2000; Sato et al., 2010). *O*-methyltransferases typically catalyze the transfer of a methyl group from *S* -adenosylmethionine (SAM) to their respective substrates. Although the substrate structures of different *O*-methyltransferases are similar, they exhibit strict substrate specificity. Comparative analysis of *O*-methyltransferase sequences from various sources reveals a highly conserved C-terminal region responsible for SAM binding, whereas the N-terminal region tends to vary. The N-terminal domain plays a pivotal role in substrate recognition, and modification of this domain can potentially alter the substrate specificity of the enzyme (Joshi and Chiang, 1998; Zubieta et al., 2001). Reticuline *N*-methyltransferase, pavine *N*-methyltransferase, and tetrahydroproberberine *N*-methyltransferase were characterized during the biosynthesis of BIAs (Kum-Boo et al., 2002; Liscombe and Facchini, 2007; Morris and Facchini, 2016; Torres et al., 2016). Currently, the successful heterologous expression of these *N*-methyltransferases serves as a useful resource for the heterologous reconstruction of BIA biosynthetic pathways.

Although more than 30 components of BIAs have been isolated from lotus, the biosynthetic enzymes associated with their production are poorly understood. In 2020, Menendez-Perdomo and Facchini reported the identification of 2 *O*-methyltransferases from *N. nucifera*, namely NnOMT1 and NnOMT5 (Menéndez-Perdomo and Facchini, 2020). The functional characterization of these recombinant proteins revealed NnOMT1 as a regiospecific 6OMT that was capable of accepting both *R* and *S* substrates. Conversely, NnOMT5 primarily acts as a 7-*O*-methyltransferase with minor activity toward 6OMT, exhibiting a strong stereospecific preference for *S*-enantiomers. Given the limited understanding of the mechanism of formation of (*R*)-enantiospecific BIAs, the protein crystal structure of NnNCS1 was determined to serve as an invaluable enzymatic tool for synthetic biology studies related to the biosynthesis of (*R*)-BIAs (Zhang et al., 2023). Multiple crystal structures of the 2 variants of scoulerine 9OMT (S9OMT) from *Thalictrum flavum* were resolved, enabling comparative analysis with the crystal structure of TfS9OMT and *T. flavum* norcoclaurine 6OMT. This analysis identified a crucial residue responsible for regional specificity and was further validated based on mutagenesis and *in vitro* experiments. Subsequently, several mutants of TfS9OMT were generated to expand the substrate range for multiple BIAs, whereas strategically designed mutants with region-specific changes were analyzed in scoulerine-producing yeast chassis cells. This led to the successful facilitation of the production of tetrahydropalmatrubine and tetrahydropalmatine (Valentic et al., 2020). Aporphine alkaloids, an important class of BIAs, are natural compounds that have a broad spectrum of pharmacological effects. Recently, the involvement of the CYP80 enzymes AcCYP80G7 and AcCYP80Q8 in the formation of the aporphine alkaloid skeleton in *Aristolochia contorta* has been reported (Meng et al., 2024).

#### 2.2.2 Berberine bridge enzyme

BBE plays a pivotal role in the biosynthetic pathway of tetrahydroproberberine alkaloids (belonging to BIAs). It catalyzes the *N*-methyl cyclization on (*S*)-reticuline, leading to the formation of the “C” ring and the production of (*S*)-scoulerine, which constitutes the fundamental framework of tetrahydroproberberine alkaloids (**Figure 2**). This enzyme has been found in plants including *Eschscholzia californica*, *P. somniferum*, and *T. flavum* (Facchini et al., 1996; Winkler et al., 2006; Zubi Liu et al., 2013). The molecular structure of BBE consists of the following 2 domains: the FAD-binding domain and the α/β-binding domain (Winkler et al., 2008). Its N-terminal region contains a signal peptide consisting of more than 20 amino acids. It localizes within vacuoles as a nontransmembrane protein. BBE targets the endoplasmic reticulum through signal peptide cleavage and is subsequently transported to vacuoles as intracavicular proteins within the intimal system. Due to the acidic conditions in vacuoles (pH lower than the optimal alkaline pH for BBE activity), BBE is inactivated upon entering the vacuoles (Bird and Facchini, 2001). Researchers have successfully achieved the functional expression of BBE in *Saccharomyces cerevisiae* and *Pichia pastoris* (Silvia et al., 2012; Galanie and Smolke, 2015). No detectable functional expression of BBE was noted in *Escherichia coli* (*E. coli*), impeding the development of the BIA biosynthetic pathways from heterologous constructs in this bacterial host (Kuroki, 1994). Although co-culturing has been used by some researchers, the upstream pathway of BBE utilizes *E.coli*, whereas BBE requires yeast for the biosynthesis of (*S*)-scoulerine. However, due to the inconvenience in cell-culturing conditions as well as the entry and exit of intermediate cells, the co-culture method is unsuitable for large-scale cultivation. The main challenge in biosynthesizing this alkaloid using a prokaryotic chassis organism lies in achieving the prokaryotic activity expression of BBE. Our team was the first to successfully achieve the active expression of the key enzyme BBE in the tetrahydroproberberine alkaloid biosynthetic pathway in a prokaryotic host by optimizing gene sources, expression tags, and vectors. This breakthrough effectively addressed the bottleneck associated with reconstructing the entire tetrahydroproberberine alkaloid biosynthetic pathway in *E. coli* (Zhao et al., 2021). This breakthrough accomplishment has laid a solid foundation for the heterobiosynthesis of BIAs using *E. coli*. Furthermore, the researchers discovered that the berberine pontozyme-like gene, which undergoes tandem replication in the *Corydalis tomentella* genome, appear to be involved in the biosynthesis of cavitine (a BIAs compound) (Xu et al., 2022b). Recently, an endoplasmic reticulum compartmentalization strategy has been developed to enhance the activity of the vacuolar protein BBE, leading to a greater than 200% increase in the production of the key intermediate (*S*)-scoulerine (Jiao et al., 2024).

#### 2.2.3 Cytochrome P450

Cytochrome P450 is mainly hydroxylated in the BIA biosynthetic pathway by NMCH (**Figure 2**) (Pauli and Kutchan, 1998; Ikezawa et al., 2003; Yang et al., 2017), cheilanthifoline synthase (Takemura et al., 2013; Yahyazadeh et al., 2017), salutaridine reductase (Geissler et al., 2007; Ziegler et al., 2009; Higashi et al., 2010, 2011), and codeinone reductase (Alcantara et al., 2005; Sharafi et al., 2013; Dastmalchi et al., 2018). When plant cytochrome P450s are heterologously expressed in prokaryotes, the absence of matching endoplasmic reticulum limits the formation of functions. Cytochrome P450 is predominantly expressed in eukaryotic host cells; however, recent studies have demonstrated the successful expression of numerous plant-derived P450 enzymes in *E. coli* by modifying these enzymes, including those involved in the BIA biosynthetic pathway (Nakagawa et al., 2011; Lin and Yan, 2012). In 2016, Minami successfully expressed the P450 enzymes STORR and SaLSN in *E. coli* by removing the transmembrane domain. They subsequently synthesized opioid alkaloids based on the step-by-step fermentation of engineered *E. coli* (Nakagawa et al., 2016). This study provides valuable insights into the heterologous prokaryotic expression of P450 enzymes in the BIA biosynthetic pathway, enabling BIA biosynthesis using prokaryotic chassis cells. The successful expression of plant-derived P450 enzymes in prokaryotic hosts therefore paves the way for the heterologous reengineering of secondary metabolic product biosynthetic pathways in prokaryotic chassis organisms. Traditionally, FAD was believed to rely on oxidase to catalyze the classical berberine-bridging activity; however, recent studies have proposed that cytochrome oxidase CYP71BG29 functions as a berberine-bridge enzyme in *Phellodendron amurense* (Xu et al., 2024b).

### 2.3 Transcriptional regulation of BIAs

The biosynthesis of plant secondary metabolites involves not only enzyme genes but also transcription factor regulation, with the latter playing a crucial role in this process (Schluttenhofer and Yuan, 2015). By modulating the expression of target genes, transcription factors can effectively promote the biosynthesis of secondary metabolites, thereby enhancing resistance to both biotic and abiotic stresses (Broun, 2004; Cao et al., 2018; Lai et al., 2019; Xu et al., 2022a). Transcription factors can modulate the biosynthesis of secondary metabolites by orchestrating complex metabolic pathways in plants from a holistic perspective (Yuan and Grotewold, 2015). To date, transcription factors have been identified as regulators of the biosynthesis of BIAs in multiple plant species (Deng et al., 2018; Huang et al., 2023; Hong et al., 2025). The PsWRKY transcription factor in *P. somniferum* interacts with the W-box, a consensus cis-element found in the promoters of BIAs pathway genes, thereby activating transcription from the tyrosine/DOPA decarboxylase (TYDC) promoter (Mishra et al., 2013). StWRKY8 regulates the BIAs biosynthetic pathway in potato, enhancing resistance to late blight (Yogendra et al., 2017). Overexpression of the *CjWRKY1* gene from *C. japonica* led to a significant increase in the accumulation of BIAs in the culture medium of *E. californica* cells (Yamada et al., 2017). CcbHLH001 and CcbHLH0002 from *C. chinensis* were found to interact with the promoters of the berberine biosynthesis pathway genes *CcBBE* and *CcCAS*, indicating their potential regulatory roles in BIA biosynthesis. In addition, researchers have also identified that the AP2/ERF transcription factors play significant roles in the biosynthetic regulation of BIAs (Zhang et al., 2024).

## 3 Heterogeneous reconstruction of the BIA biosynthetic pathways

BIAs are mainly extracted from medicinal plants and have a wide range of pharmacological activities. With the exception of some BIAs such as berberine in *Coptis coptis* and tetrahydropalmatine in *Stephania intermedia*, the content of most BIAs in medicinal plants is relatively low (Zhao et al., 2020). Therefore, several scholars have studied them after their biosynthesis. Galanie et al. introduced 7 BIA biosynthesis–related genes from different plants into *Saccharomyces cerevisiae* and achieved berberine biosynthesis through enzyme-mutation screening, gene copy number optimization, intermediate addition, and culture condition optimization. Furthermore, the biosynthesis of opioid (thebaine and hydrocodone) alkaloids has now been achieved (Galanie et al., 2015).

### 3.1 Heterogeneous reconstruction of the BIA biosynthetic pathways using *E. coli* as the host organism

The genetic background of *E. coli* is well established and encompasses a diverse range of suitable strains and various vectors. Moreover, *E. coli* has been successfully used to express numerous eukaryotic genes at high levels. Additionally, the simplicity of operating and culturing *E. coli*, along with its cost-effectiveness and lower requirements, further contribute to its advantages (Lee and Lee, 2003). Recently, there have been numerous reports on the biosynthesis of BIAs using *E. coli*. Fumihiko Sato employed the *E. coli* fermentation system to biosynthesize (*S*)-reticuline (55 mg/L), a crucial intermediate for BIAs, using dopamine as a substrate. The co-culture of genetically engineered strains of *E. coli* and *S. cerevisiae* resulted in the production of magnoflorine and (*S*)-scoulerine at yields of 7.2 mg/L and 8.3 mg/L, respectively (Minami et al., 2008). Furthermore, using *E. coli as* the base organism, this research group initiated the biosynthesis of the key BIA intermediate (*S*)-reticuline starting from L-tyrosine, resulting in a good yield of 46 mg/L (Nakagawa et al., 2012). Next, the biosynthesis of (*R,S*)-tetrahydropapaveroline was achieved in *E. coli* using glycerol as a starting material (Nakagawa et al., 2014). Subsequently, this team accomplished the step-by-step fermentation synthesis of thebaine in *E. coli* using glycerol as a starting material, resulting in a yield of 2.1 mg/L. However, there are challenges associated with the transmembrane transport of intermediates between these 2 microorganisms and scaling up their production via fermentation using the co-culture approach. Our team identified the biosynthesis-related genes of tetrahydroproberberine from plants and microorganisms. Subsequently, *E. coli* was used as a host to reconstruct the entire biosynthetic pathway, overcoming the bottleneck in the biosynthesis of tetrahydroproberberine using *E. coli*. By implementing a modular biosynthesis strategy, 5 synthetic modules for tetrahydroproberberine alkaloids were successfully constructed and introduced into *E. coli* to generate multiple engineered strains that were capable of producing tetrahydroproberberine, and corydamine (Zhao et al., 2021). Subsequently, directed methylation biosynthesis of tetrahydroproberberine compounds was achieved by combining different *O*-methyltransferases (Zhao et al., 2023).

### 3.2 Heterogeneous reconstruction of the BIA biosynthetic pathway in yeast

The yeast system has been used extensively for the heterologous reconstruction of secondary biosynthetic pathways of plant metabolites. Yeast systems have well-defined metabolites and genetic backgrounds (Borodina and Nielsen, 2014; Yunzi et al., 2015). Moreover, compared with bacterial systems, yeast exhibits a microenvironment more akin to plant metabolism, enabling the posttranslational processing and modification of proteins that contribute to the expression of plant-derived membrane proteins. There are numerous reports on the expression of single enzymes or multiple synthases using yeast systems (Ikezawa et al., 2007; Deloache et al., 2015; Trenchard et al., 2015; Hori et al., 2016; Chen et al., 2018; Farrow et al., 2018; Dastmalchi et al., 2019; Ping et al., 2019; Srinivasan and Smolke, 2019). Although *E. coli* is routinely used to express plant-derived P450 enzymes, it is generally acknowledged that yeast facilitates the relatively facile expression of plant-derived membrane proteins. Hawkins and Smolke introduced various enzymes including 6OMT, CNMT, 4′OMT, SMT, and BBE from plants and CYP2D6 from humans into the chassis organism *S. cerevisiae* through different combinations. Using (*R,S*)-norrhodanine as a substrate, the team achieved yields of 32.9 mg/L of (*R,S*)-reticuline, 60 mg/L of (*S*)-tetrahydrococlumbamine, 30 mg/L of (*S*)-tetrahydroberberine, and 20 mg/L of salutaridine (Hawkins and Smolke, 2008). Furthermore, the biosynthesis of stylopine, cis-*N*-methylstylopine, protoopine, and sanguinarine was achieved using (*R,S*)-norlordanine as a substrate (Trenchard and Smolke, 2015). In the same year, the research team expressed the enzymes involved in multiple BIA biosynthetic pathways in yeast, and successfully biosynthesized thebaine (expressing 21 enzymes) and hydrocodone (expressing 23 enzymes) using glucose (Galanie et al., 2015). In 2018, Smolke et al. successfully integrated more than 30 genes sourced from diverse organisms including plants, animals, bacteria, and yeast into yeast chassis cells. They successfully achieved the *de novo* biosynthesis of noscapine, an opioid derivative, via highly optimized (*S*)-reticuline biosynthesis. By optimizing key enzymes and using suitable strategic approaches, a high yield of 2.2 mg/L of noscapine was obtained when this fermentation process was used (Li et al., 2018b). Jamil et al. successfully biosynthesized tetrahydropapaverine in yeast and obtained the drug papaverine using semisynthetic methods, indicating a novel and alternative synthetic approach (Jamil et al., 2022). Qishuang Li et al. successfully achieved the *de novo* biosynthesis of magnoflorine in yeast using glucose as a substrate, resulting in a yield of 75.8 mg/L (Li et al., 2024). However, the heterologous biosynthetic yield of alkaloids is relatively low and can be attributed to the complex, lengthy, biosynthetic pathways that involve numerous enzymatic reactions and enzymes. For example, the biosynthetic pathway of noscapine involves more than 30 enzymatic reactions, posing a challenge to optimize its expression and culture conditions. Recent studies have demonstrated biocatalytic cascades to biosynthesize (*S*)-norcoclaurine (Zhao et al., 2022) and methylated tetrahydroprotoberberine and protoberberine alkaloids (Roddan et al., 2021), highlighting the suitability of alternative routes for BIA biosynthesis.

### 3.3 Biosynthesis of BIAs in plants

The biosynthetic pathways of most BIAs are highly intricate and involve membrane-binding proteins, thereby necessitating specialized intracellular infrastructure. Transferring such mechanisms for component biosynthesis to other organisms is therefore challenging. Compared with microbial hosts, exogenous plant hosts offer microenvironments and core metabolic pathways that closely resemble those of the original host plants in terms of exogenous protein expression. Therefore, the latter serve as natural chemical precursors for downstream biosynthesis pathways. A limited number of model plants are currently being used to engineer chassis organisms to synthesize natural products. The Tobacco BY-2 cell line, derived from tobacco seedlings, has been extensively utilized in cell suspension culture and serves as a model organism to study the molecular biology and physiology of plants. Moreover, this cell line is susceptible to several plant viruses, rendering it an ideal model for studying plant–pathogen interactions. Additionally, the utilization of tobacco has facilitated the reprogramming of various biosynthetic pathways involving compounds such as sesquiterpenes, diterpenes, monoterpenes, lignans, and glucosinolates via the incorporation of up to 10 enzymes (Wu et al., 2006; Lau and Sattely, 2015; Kotopka et al., 2018). Moreover, nonmodel plants can be selected as foreign hosts for the biosynthesis of natural products. A primary reason for utilizing a nonmodel plant host is the presence of a specific upstream substrate in the chosen organism. For instance, *Artemisia annu*a has been used to biosynthesize taxadiene owing to its abundant terpene precursors (Li et al., 2015). *A. annu*a grows rapidly and has an efficient genetic transformation system that can be converted into functional expression heterologous plant enzymes. The establishment of hairy root cultures and genetic transformation provides a robust foundation for the production of BIAs using plant chassis (Zhang et al., 2023). However, allogenic plant systems, such as native plants, have slower growth rates compared to microbial hosts and often lack convenient and stable genome engineering systems. Moreover, they demonstrate a complex metabolite background that may contain compounds similar to the target compounds, thereby introducing challenges in isolating the required components.

## 4 Research on the biosynthesis strategies of BIAs

Reconstruction of biosynthetic pathways is met with numerous challenges due to the complex nature of certain natural products and their associated biosynthetic pathways. Synthetic biology approaches have been developed to overcome these obstacles. Some such approaches include precise regulation of foreign gene expression, enhancement of the functional expression of foreign enzymes, and modification of central metabolism to augment the entry routes of precursor compounds. These strategies have been used to promote pathway-reconstruction approaches for plant-based natural products. Furthermore, elucidation of biosynthetic pathways in plants that are not currently well understood is being attempted via enzyme mining or engineering by using both natural and nonnatural hosts. By enhancing efficiency, selectivity, expression levels, or electron-transfer capabilities, the activity of plant-derived enzymes—particularly that of cytochrome P450—is bolstered. The overall reaction efficiency in multienzyme pathways can be enhanced via dynamic control by partitioning or optimization of host metabolism (Li et al., 2018a). To enhance the efficiency of multienzyme co-expression in a pathway, the synthesis of natural product components using microbial chassis involves a diverse array of endogenous metabolic networks that orchestrate intracellular reactions. Consequently, pathway efficiency is influenced by both intra- and extracellular interactions between metabolites and enzymes. To optimize pathway efficiency, researchers have devised numerous strategies such as the dynamic modulation of enzyme expression levels in the pathway, compartmentalization of enzymes to facilitate interactions with metabolites in the pathway, and the global optimization of heterologous biosynthesis pathways utilizing endogenous microbial metabolic networks.

## 5 Summary and future prospects

Recent advances in technology have increased the feasibility of utilizing microorganisms to biosynthesize secondary plant metabolites having complex structures and high value. Heterologous biosynthesis platforms reported to date demonstrate the possibility of achieving high yields and support the expression of numerous heterologous enzymes involved in complex biosynthetic pathways. The integration of genomics, bioinformatics, and synthetic biology has significantly facilitated the discovery of secondary plant metabolites and their potential biosynthetic routes. Despite rapid progress in sequencing data analysis in the fields of plant genomics and functional genomics, inherent trade-offs between data quality, scale, and measurement speed persist. A systematic interface between computational research and experimental science is therefore essential to bridge the gap between the transitioning of raw data to valuable knowledge. Future developments encompassing annotated information related to plant genomics, transcriptome analysis associated with plant secondary metabolism, and microbial and plant biosynthetic pathway reconstruction can be integrated into a comprehensive pipeline for microbial biosynthesis platforms that will advance microbial engineering toward synthesizing complex secondary plant metabolites while enhancing our understanding of the intricate protocols that govern natural product biosynthesis.

BIAs are crucial in advancing medical and societal domains; however, their availability is often limited by the scarcity of plant resources. Recent advances in synthetic biology have elucidated the biosynthetic pathways and enzymes involved in the biosynthesis processes of several alkaloids. Heterologous reconstruction of BIA biosynthetic pathways in bacteria and yeast enables a cost-effective production of complex active ingredients or drugs using inexpensive starting materials. Nevertheless, the biosynthesis of BIAs is met with several challenges. First, most biosynthetic pathways for BIAs, particularly those involving complex structures, are poorly understood, posing a challenge in identifying and transforming highly efficient synthases within these metabolic routes. Second, although various enzymes involved in BIA biosynthetic pathways have been successfully expressed in heterologous hosts (including membrane-binding proteins), the current levels of production and demands cannot meet the high industrial requirements due to factors such as low enzyme expression and difficulties in optimizing metabolism using engineered strains. Therefore, further developments in synthetic biology are warranted for the discovery of innovative solutions—particularly through cross-disciplinary integration with multiomics technologies—to enable the large-scale biosynthesis of trace natural products.
